# Knowledge Visualizations to Inform Decision Making for Improving Food Accessibility and Reducing Obesity Rates in the United States

**DOI:** 10.3390/ijerph17041263

**Published:** 2020-02-16

**Authors:** Raphael D. Isokpehi, Matilda O. Johnson, Bryanna Campos, Arianna Sanders, Thometta Cozart, Idethia S. Harvey

**Affiliations:** 1 Center for Trans-Disciplinary Data Analytics, Department of Natural Sciences, College of Science, Engineering and Mathematics, Bethune-Cookman University, Daytona Beach, FL 32114, USA; arianna.m.sanders@students.cookman.edu; 2 Department of Public Health and Health Equity, Petrock College of Health Sciences, Bethune-Cookman University, Daytona Beach, FL 32114, USA; johnsonma@cookman.edu (M.O.J.); bryanna.a.campos@students.cookman.edu (B.C.); cozartt@cookman.edu (T.C.); 3 Health Equity Internship Program, Association of State Public Health Nutritionists, PO Box 37094, Tucson, AZ 85740, USA; 4 Transdisciplinary Center for Health Equity Research, Department of Health and Kinesiology, Texas A & M University, College Station, TX 77843, USA; isharvey@tamu.edu

**Keywords:** food access, Food Access Research Atlas (FARA), food desert, knowledge visualization, obesity, visual analytics

## Abstract

The aim of this article is to promote the use of knowledge visualization frameworks in the creation and transfer of complex public health knowledge. The accessibility to healthy food items is an example of complex public health knowledge. The United States Department of Agriculture Food Access Research Atlas (FARA) dataset contains 147 variables for 72,864 census tracts and includes 16 food accessibility variables with binary values (0 or 1). Using four-digit and 16-digit binary patterns, we have developed data analytical procedures to group the 72,684 U.S. census tracts into eight and forty groups respectively. This value-added FARA dataset facilitated the design and production of interactive knowledge visualizations that have a collective purpose of knowledge transfer and specific functions including new insights on food accessibility and obesity rates in the United States. The knowledge visualizations of the binary patterns could serve as an integrated explanation and prediction system to help answer why and what-if questions on food accessibility, nutritional inequality and nutrition therapy for diabetic care at varying geographic units. In conclusion, the approach of knowledge visualizations could inform coordinated multi-level decision making for improving food accessibility and reducing chronic diseases in locations defined by patterns of food access measures.

## 1. Introduction

### 1.1. Overview

In the context of a community-based food system, research on food access is in the dimensions of availability, accessibility, affordability, acceptability, and accommodation [1,2,3]. In particular, food accessibility is the geographic location of the food supply and ease of getting to that location [2,3]. Measures for food access within geographic units are provided at the individual and area levels [4]. Data at the geographic unit of the census tract in the United States are available for some food access measures through the United States Department of Agriculture (USDA) Food Access Research Atlas (FARA) [5,6]. A census tract is a small statistical subdivision of a county that usually contains between 1200 and 8000 people but generally averages approximately 4000 people [7,8]. In the context of public health, knowledge creation activities include research and development, solving a public health problem, devising a public health promotion strategy, discovering a pattern, developing a public health program or intervention, or conducting monitoring and evaluation activities [9]. These public health activities result in complex public health knowledge for education, research, and practice. 

In the knowledge visualization process, visual representations are designed to communicate experiences, insights and potentially complex knowledge [10]. The growing infusion of complex, large, interactively accessed, professionally collected datasets in education at undergraduate, graduate and professional levels presents challenges to the design of effective instruction and learning strategies [11,12,13]. Furthermore, effectively designed knowledge visualizations help to reduce decision maker’s information overload, misinterpretation, misuse, underutilization or inability to use information [14]. Thus, the aim of this article is to promote the use of knowledge visualization frameworks in the creation and transfer of complex public health knowledge. The overall goal of the research project reported here is to support knowledge creation and knowledge transfer on the relationship between food accessibility and chronic diseases in the United States. The chronic disease of interest in this report is obesity. The following sections introduce knowledge visualization, the Food Access Research Atlas, and the research objectives.

### 1.2. Knowledge Visualization for Knowledge Creation Activities in Public Health

We focus in this research article on pattern discovery from public health-relevant datasets. The methods for knowledge transfer facilitate effective communication of complex analytic results and uptake of relevant information for policy and priority considerations [15]. Knowledge visualization supports knowledge creation and knowledge transfer through a framework to design visualizations of datasets that have cognitive (attention, recall, elaboration and new insights), emotional (motivation) and social (coordination) benefits [16]. The functions of knowledge visualization are (1) coordination (coordinate the communication of knowledge workers); (2) attention (raise awareness and provide focus for knowledge creation and transfer); (3) recall (improve memorability and thus foster the application of new knowledge); (4) motivation (energize viewers to engage in interpretation and explore the graphic); (5) elaboration (the process of visualizing knowledge leads to further understanding and appreciation of concepts and ideas as one interacts with them); and (6) new insights (knowledge visualizations can reveal previously hidden connections and lead to sudden insights, a-ha experiences). 

The visualization types in knowledge visualization are sketch, diagram, image, map, object, interactive visualization and story [16]. Our primary visualization type is interactive visualization, which can be accomplished with visual analytics software. Thus, our approach to constructing knowledge visualizations involves constructing enhanced visualization-ready datasets and interactive visualizations that provide cognitive, social and emotional functions. The Data Visualization Literacy Framework (DVL-FW) presents the typology and the process for constructing and interpreting data visualizations, which is relevant to our research [17]. We present more details of the DVL-FW and other frameworks in the overview section of the Materials and Methods of this article. 

### 1.3. Food Access Research Atlas Dataset for Public Health Knowledge Creation 

The USDA Economic Research Service (ERS) Food Access Research Atlas dataset is currently the most comprehensive food environment classification at the census tract geographic entity in the United States [18]. We view the FARA dataset as a complex analytics result that needs to be effectively communicated to guide policy making as well as a data source for public health knowledge creation. Thus, the communicative power of knowledge visualizations [10] is a consideration in our research. The FARA dataset has several advantages despite that the food access measures emphasized are household income, distance to supermarket, and availability of the household vehicle. The Food Access Research Atlas (1) provides standard measures at the census-tract level that can be used across the United States; (2) is widely used by government policy makers to guide policy interventions to improve food access; and (3) can facilitate the understanding of obesity at the population level [19,20]. The FARA dataset serves as a source for identifying neighborhoods for research on diet-related health outcomes including incident cardiovascular events [21], risk of pre-term birth for underweight women [22], and glycemic control in patients with diabetes [23]. 

### 1.4. Need for Knowledge Visualizations to Communicate Knowledge on Food Access Measures in the Food Access Research Atlas Dataset

The USDA FARA dataset (May 2017 update) (referred to here as the FARA dataset) consist of 72,864 census tracts and 147 variables including new variables on total census tract counts of subpopulations [5]. The USDA FARA dataset contains both categorical and continuous variables that are suitable for identifying patterns and subgroups of geographical units including census tracts. Each census tract record in the FARA dataset includes 16 variables with binary values (0 or 1) that describe urban/rural status, presence of group quarters as well as flags for measures of income, distance to food supply (nearest supermarket, supercenter, or large grocery store) and availability of vehicle to household. The FARA dataset includes four measures that combine low income (LI) and low access (LA) to capture the food desert status of a census tract [7]. The food desert (LILA) measures are (1) low-income and low-access tract measured at 1 mile for urban areas and 10 miles for rural areas; (2) low-income and low-access tract measured at 1/2 mile for urban areas and 10 miles for rural areas; (3) low-income and low-access tract measured at 1 mile for urban areas and 20 miles for rural areas; (4) low-income and low-access tract using vehicle access or low-income and low-access tract measured at 20 mile A census tract described as a food desert has at least 33 percent of the tract’s population or a minimum of 500 people in the tract must have low access to a supermarket or large grocery store [5].

Therefore, our first research objective was to design and implement knowledge visualizations that communicate census tracts in the FARA datasets as subgroups defined by binary numbers. Two sets of binary numbers (16-digit and 4-digit) were constructed to respectively describe the status of food access measures and the food desert measures. The inclusion of these binary number patterns as fields in the FARA dataset can expand the type of data analytics techniques (including visual analytics; statistical analysis; and modeling and simulation) that researchers can apply to further understand the interactions of food access measures in communities. Binary datasets represent a compact and simple way to store data about the relationships between a group of objects and their possible properties [24].

### 1.5. Need for Knowledge Visualizations to Communicate Knowledge on Adult Obesity Rates in Census Tracts with Identical Food Access Measures 

Access to food that is healthy and nutritious is a factor that influences the prevalence of obesity [25,26,27]. Obesity is a controllable risk factor for type 2 diabetes, which disproportionately affects rural Americans [28]. The 2006–2010 prevalence estimates of adult obesity rates for the conterminous U.S. census tracts and ZIP codes in 2399 counties have been estimated from the Centers for Disease Control and Prevention’s Behavioral Risk Factor Surveillance System (BRFSS) [29]. The BRFSS provides “precise regional estimates of obesity prevalence that are very valuable for tracking temporal changes in obesity rates” [30]. 

The availability of data on adult obesity rates for 2620 census tracts in the Commonwealth of Pennsylvania provided an example dataset to coordinate the knowledge between food accessibility and obesity rates in census tracts of the United States. We expected that a subgroup defined by a pattern of food access measures would contain census tracts with a range of adult obesity rates. Thus, our second research objective was to design and implement knowledge visualizations of census tract subgroups with the same pattern of food access measures but significantly different obesity rates. The subgroups of census tracts could be the basis for research on factors in the food access dimensions that influence differential obesity rates between census tracts of identical patterns of food access measures.

## 2. Materials and Methods 

### 2.1. Overview

The materials and methods reported in this article build on our prior research approaches using visual analytics techniques and technologies [31,32,33,34]. Since the knowledge visualization type of interest is interactive visualization, the methods of the research project are broadly divided into (1) the construction of datasets and (2) the design and implementation of interactive worksheet views and dashboards. The methods use techniques for addressing the data challenges dimensions of data flow (i.e., collection, storage, access, and movement), data analytics (i.e., modeling and simulation, statistical analysis, and visual analytics), and data curation (i.e., preservation, publication, security, description, and cleaning) [35]. We constructed datasets with appropriate software such as those for scripting, database management, spreadsheet, statistical analysis, and visual analytics. 

The design and implementation of interactive views of datasets were performed in software for visual analytics following guidelines on (1) interaction design for complex cognitive activities [12,36,37]; (2) knowledge visualization [10,38,39,40,41]; (3) knowledge generation model for visual analytics [42]; (4) six categories of visuals for easy construction and interpretation of visuals [43]; (5) data visualization literacy framework (DVL-FW) [17]; (6) how visual representations are likely to affect the decision processes or tasks [44]; and (7) mindful engagement with visuals for optimal decisions [45]. The last three frameworks are particularly relevant to decisions on the core concept types of visualizations as well as the process of constructing and interpreting visualizations. 

Visuals are a form of language that impact cognition, requiring humans to have experiences and familiarity with visuals to make optimal decisions using visuals [45]. To construct and interpret visuals at a basic level, visuals can be categorized into six categories: one dimensional (e.g., box plot), two dimensional (e.g., bar graph), map (e.g., street map), shape (e.g., pie chart), connection (e.g., flow chart) and picture (e.g., pattern) [43]. The data visualization framework or theory (DVL-FW) provides the core visualization concept types (insight needs, data scales, analyses, visualizations, graphic symbols, graphic variables and interaction and the core process stages for constructing and interpreting data visualizations. The stages in the core visualization process are (1) Stakeholders: identification of stakeholders and their insight needs; (2) Acquire: acquisition of relevant datasets and other resources to address the well-defined insight needs; (3) Analyze: preprocessing of datasets through analysis; (4) Visualize: selection of visualization type, graphic symbols and graphic variables for the dataset; (5) Deploy: deploying the visualization in interaction types; and (6) Interpret: the interpreting stage of reading and translating the visualization into insights of real world application [17]. The framework for thinking about how visual representations are likely to affect the decision processes or tasks consists of the visual perspective and information context and associated 24 propositions [44]. A proposition that is applicable to interactive visualization type is that compared with non-interactive displays, interactive visualization tools lead to more accurate decisions. Furthermore, cognition of data is positively influenced by use of purposefully designed visuals [45]. Thus, the knowledge visualizations that we sought to produce are purposeful and constructed to be interactive to facilitate more accurate decisions [43,45]. The justifications for the selection of specific core data visualization concept types and core data visualization process are provided where needed for the knowledge visualizations constructed.

The remaining description of the materials and methods section is:

(1) Data Source for Constructing Food Access Patterns for Census Tracts in the United States.
(2) Construction of Food Access Patterns using Variables with Binary Values (0 or 1).
(3) Design and Implementation of Interactive Visualizations as Solutions for Grouping Census Tracts by Food Access Patterns.
(4) Comparison of Obesity Rates in Census Tracts.

### 2.2. Data Sources for Constructing Food Access Patterns for Census Tracts in the United States

**Figure 1:**
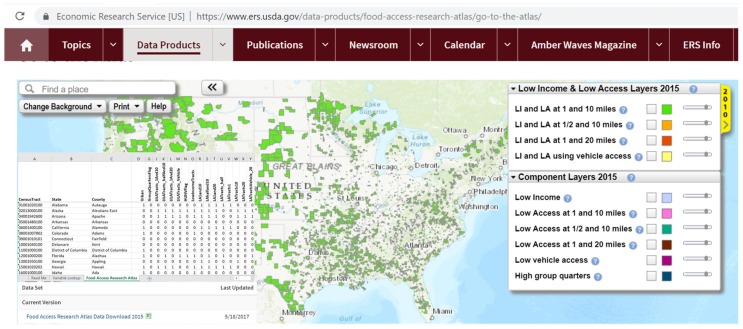
Data sources for constructing the 16-digit binary number encoding food access measures. Section of the Food Access Research Atlas spreadsheet (placed on map) shows the 16 variables combined to form the food access pattern for each census tracts. The boxes showing the legend to the layers of the map has ten of the variables.

The datasets associated with the United States Department of Agriculture’s (USDA) Food Access Research Atlas (FARA) were the data sources for constructing the patterns of food access measures for census tracts in the United States (Figure 1). A spreadsheet workbook file (version 18 May 2017) consisting of three worksheets [36] (Read Me, Variable Lookup and Food Access Research Atlas) is available on the website of the USDA’s Economic Research Service (Figure 1). The Read Me worksheet provides notes about the Food Access Research Atlas download data. 

The Variable Lookup worksheet has three columns (Field, LongName and Description of variables) with a header row and 147 rows of records. The Food Access Research Atlas worksheet has entries in 147 columns and 72,865 rows including a header row. This multivariate dataset has the Census Tract identifier (an 11-digit numeric) as the unique identifier for each record. The variables are categorized as (1) general tract characteristics, (2) low-access and distance measures, (3) vehicle availability, (4) group quarters, (5) low-income and low-access measures, and low access by population subgroups [5]. Examples of demographic variables are state name, county name, population count from 2010 census, and occupied housing unit count from the 2010 census.

### 2.3. Construction of Food Access Patterns Using Variables with Binary Values (0 or 1)

The Food Access Research Atlas (FARA) dataset includes 16 variables that have “flag” in their descriptions. This “flag” label indicates a variable with a binary value of 0 or 1 (Table 1). We uploaded the FARA dataset in visual analytics software, Tableau Desktop Professional (Tableau Software Inc., Seattle, WA, USA). Subsequently, we defined two calculated fields to concatenate the binary values (coded as a string data type in Tableau) and generated a pattern assigned to each United States census tract. The 16-digit binary number (Food Access Pattern) encodes the values for all the 16 FARA “flag” variables. Thus, a pattern of “1000000000000000” indicates an urban census tract that is not in a food desert, not having a high share of group quarters, not having the LILA or LA variables; not a low-income tract and having high vehicle access.

The four-digit binary number (Food Desert Pattern or LILA Pattern) encodes the values for the four low-income low-access (LILA) variables. Thus, a pattern of “1111” indicates the presence of all the LILA variables in a census tract. The additional two fields were inserted after the Census Tract field in the original spreadsheet file to become a Food Access Research Atlas dataset annotated with food access patterns and food desert (LILA) patterns. 

### 2.4. Design and Implementation of Interactive Visualizations as Solutions for Grouping Census Tracts by Food Access Patterns

We sought to group census tracts by the 16-digit binary number profile at geographic units of national, regional, state, and county. We also constructed a dataset of environmental protection regions and counties for states. This environmental region-county dataset can be integrated with other datasets such as rates of chronic diseases to support regional decision making integrating food access measures and environmental parameters. We implemented enclosure diagrams, box plots, and geographic maps as common visualizations that support coordination, attention, recall, motivation, elaboration and new insights [16,37]. When working with geographic information, maps facilitate better decision making and problem solving [37]. Furthermore, enclosure diagrams such as tables are useful for representing precise and indexical information, both quantitatively and qualitatively. Enclosure diagrams support “decision-making by constraining the set of alternatives that one must consider during a decision-making activity and specifying paths and commonalities among different problem states within and information space” [37]. 

We have developed knowledge visualizations that combined enclosure table and bar graph because “decision makers using visual representations that include graphic and tabular information show higher performance in terms of decision accuracy and speed than decision makers using either graphic or tabular formats alone [44]. In the case of maps, the color saturation level of the graphic symbol or the size of the graphic symbol representing the geographic unit is an indication of the value of the variable projected on the map [46]. The box plots were selected to compare the distributions of specific binary patterns and to identify outliers. When multiple food access patterns are compared for a county, then each point representing the food access pattern on the box plot is assigned a color to attract attention and support comparisons among other functions [46]. Additionally, we used the boxplot and bar graph because “decision makers using graphic versus text-based (tabular) presentations of the same information are more quickly identify outliers, trends, and patterns of covariation between variables” [44]. 

In the visual analytics software (Tableau Desktop Professional), we uploaded relevant datasets and designed views with the layout of data fields for the appropriate interactive visualizations. Interaction features (such as drop lists) were included in the designs to support decision making based on data fields (Food Access Pattern, Public Health Region, State and County). We also designed dashboards consisting of views and other dashboard objects (e.g., web page, image, text and external software) to integrate views.

### 2.5. Comparison of Obesity Rates in Census Tracts

According to the Centers for Disease Control and Prevention (CDC), an estimate of the age-adjusted percentage of persons age 20 and older who are obese, where obesity is Body Mass Index (BMI) greater than or equal to 30 kg per meters squared. We obtained data on the estimate of obesity rates (2006 to 2010) for census tracts in the 67 counties of the Commonwealth of Pennsylvania from the data catalog of the website DATA.GOV. We applied the dataset on estimates of adult obesity rates for census tracts as an example to develop new insights through knowledge visualizations for identifying census tracts with significantly different obesity rates but with the same pattern of food access. The 2006–2010 estimates are expected to range from 0 to 1. Thus, we removed a census tract with value outside the range of 0 to 1 before uploading of the dataset to the visual analytics software (Tableau Desktop Professional). 

In the visual analytics software, we designed a view that integrates the data fields from multiple datasets: the FARA enhanced dataset (Food Access Pattern, County) and PAobesity dataset (CensusTract, State and 2006–2010 Estimate of Obesity). The new dataset constructed consisted of fields Census Tract, County, Food Access Pattern, State, 2006–2010 Estimate of Obesity (for each census tract). The Census Tract data field was the index field linking both datasets. To enable regional comparisons of data, we constructed a dataset with fields for the environmental regions (obtained from the website of the Pennsylvania Department of Environmental Protection) and the 67 counties in Pennsylvania. We designed a box plot view that includes details for county and region to address our objective to support the identification of census tracts with significantly different obesity rates but the same food access pattern. This type of view can display groups of census tracts according to patterns of food access further grouped with or without the county name. The first is to allow statewide comparison and the second view is for within-county comparison. Both views allow for the comparison of census tracts with the same pattern of food access measures by obesity rates. 

## 3. Results

### 3.1. Overview

**Figure 2:**
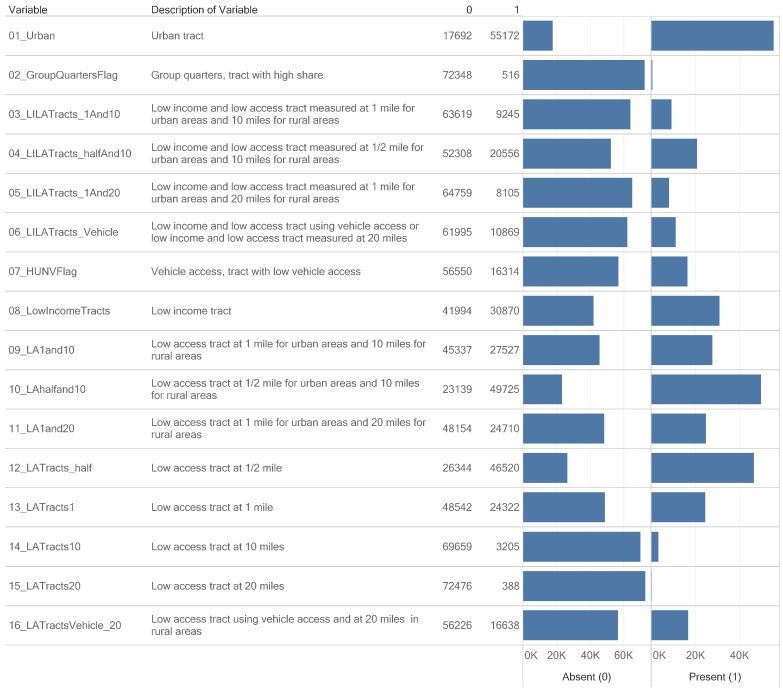
Comparison of counts of census tracts for 16 variables encoding food access measures. The knowledge visualization integrates enclosure table and bar graph to support attention and new insights on the counts of census tracts in two categories of presence or absence of the variable. Variables 02_GroupQuartersFlag and 15_LATracts20 have less than 1000 census tracts.

The results of the research investigation include dataset knowledge visualizations (available as interactive views and dashboards), findings, prior knowledge, and hypotheses for future research. The interactive knowledge visualizations of the datasets constructed are at the website of Tableau Public (provided as a supplementary information). We developed a knowledge visualization to provide an overview of the absence (0) or presence (1) of the 16 variables in the 72,684 census tracts (Figure 2). Variables GroupQuartersFlag and LATracts20 (Low-access tract at 20 miles) are present in less than 1000 census tracts with values of 516 and 388 census tracts respectively. 

According to the framework for knowledge visualization [16], we were able to gain new insights (previously hidden connections and lead to sudden insights, a-ha experiences) from Figure 2 with an attention (raise awareness and provide on focus for knowledge creation and transfer) on variables with census tract count equal or less the 1000 when variable is present. A new insight is that 516 census tracts were flagged for high share of group quarters. Additionally, 388 census tracts had the designation of the low-access tract at 20 miles, defined as “a rural tract with at least 500 people, or 33 percent of the population, living more than 20 miles from the nearest supermarket, supercenter or large grocery store.” Therefore, a prediction from this knowledge visualization based on the definition of the low-access tract at 20 miles is that the 388 census tracts will be rural census tracts. 

### 3.2. Groups of Census Tracts by Food Access Patterns

#### 3.2.1. Knowledge Visualization for Knowledge Transfer on Counts of Census Tracts in Rural and Urban Locations

The binary encoding approach classified the 72,684 census tracts in the Food Access Research Atlas into forty (40) 16-digit and eight (8) 4-digit binary numbers (Figure 2). The binary numbers serve as codes for describing the patterns of food access measures. The range of count of census tracts in each group was from 1 to 13,268. The group of census tracts with pattern 1000000001010000 had 13,268 census tracts. This census tract with the highest number is an urban census tract (first binary digit) without group quarters (second binary digit) and flagged for (1) Low-access tract at 1 mile for urban areas and 20 miles for rural areas (LAhalfand10, 10th binary digit); (2) Low-access tract at 1/2 mile (LATracts_half, 12th binary digit). 

**Figure 3:**
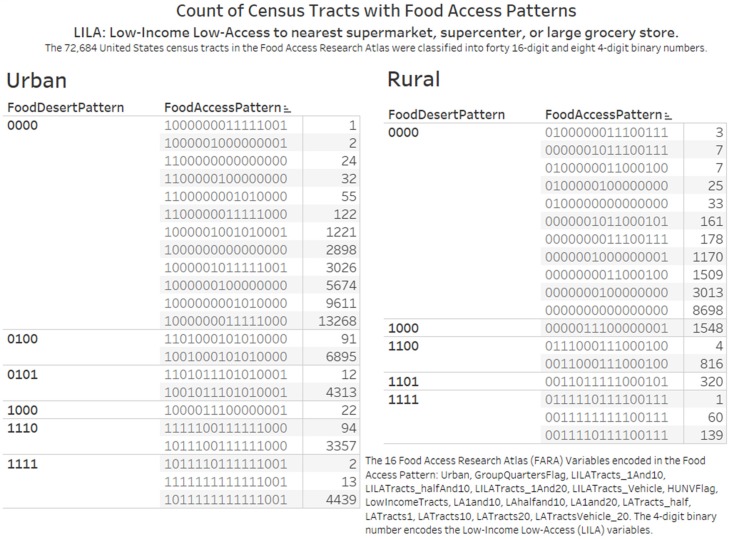
Groups of census tracts in the United States according to patterns of food access measures. This knowledge visualization provides knowledge transfer and other functions on counts of census tracts in rural and urban locations. The 16 Food Access Research Atlas (FARA) Variables encoded in the Food Access Pattern: Urban, GroupQuartersFlag, LILATracts_1And10, LILATracts_halfAnd10, LILATracts_1And20, LILATracts_Vehicle, HUNVFlag, LowIncomeTracts, LA1and10, LAhalfand10, LA1and20, LATracts_half, LATracts1, LATracts10, LATracts20, LATractsVehicle_20. The 4-digit binary number encodes the Low Income Low Access (LILA) variables. The abbreviations of the FARA variables are defined in Table 1.

In Figure 3, the knowledge visualization compares the distribution of the 4-digit LILA (low-income low-access) census tracts by rural and urban status. The 4-digit and 16-digit binary patterns allowed us to give attention to several aspects of food access measures leading to elaboration (further understanding and appreciation of concepts and ideas).

Firstly, in subgroups of the 4-digit pattern with two to three 16-digit patterns, we could identify specific variables that differentiate the census tracts. For example, in the rural subgroup with LILA pattern “1111”, the variables GroupQuartersFlag and HUNVFlag differentiate the census tracts.

Secondly, digit 15 (low-access tract at 20 miles) in the 16-digit pattern for all the urban census tracts is 0 while for rural tracts it can be 0 or 1. All the three 16-digit patterns in the rural LILA pattern of “1111” (food deserts) also had a value of 1 (presence of low-access tract at 20 miles) in digit 15. We observed 8698 census tracts with the pattern of 0000000000000000 (All zeros) indicating Rural Census Tract without Group Quarters and not flagged for Food Access Measures. Table 2 and Table 3 list the percentage of census tracts in a state with the all zeros pattern. The percent column provides the percentage of all zeros count for regional comparison. We obtained the extent of coverage of potentially food-secure rural census tracts in comparison to the census tract count. The State of Vermont in US Health and Human Services Region 1 has 184 census tracts and 91 (49.46%) have the all zeros pattern (Table 2). Of the 4214 census tracts in Florida, 271 (6.43%) have the all zeros pattern. 

#### 3.2.2. Knowledge Visualization for Elaboration of the Distribution of Food Access Measures by States

**Figure 4:**
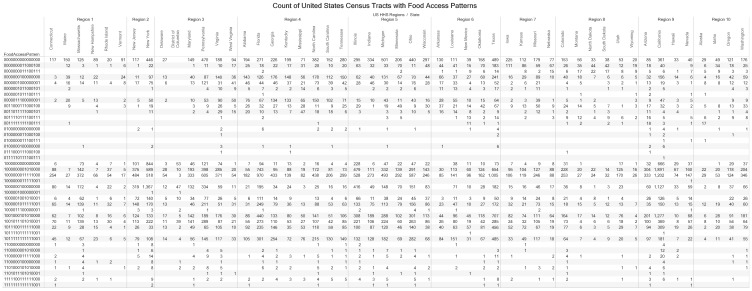
Count of United States census tracts with food access patterns grouped by United States Health and Human Services Regions. We developed a knowledge visualization (enclosure diagram) to transfer knowledge on the overview of census tracts based on counts of the 40 16-digit food access patterns. Binary numbers starting with 1 indicates urban census tract while 0 indicates rural census tract. The grouping of states by 10 U.S. HHS regions is to facilitate decision making especially by researchers interested in comparative analysis of food access measures. The online version of the visualization allows user to view records on census tracts that constitute the count (elaboration and motivation function). The digits in the Food Access Pattern encode 16 variables in the Food Access Research Atlas: Urban, GroupQuartersFlag, LILATracts_1And10, LILATracts_halfAnd10, LILATracts_1And20, LILATracts_Vehicle, HUNVFlag, LowIncomeTracts, LA1and10, LAhalfand10, LA1and20, LATracts_half, LATracts1, LATracts10, LATracts20, LATractsVehicle_20. The 4-digit binary number encodes the Low-Income Low-Access (LILA) variables. The abbreviations of the FARA variables are defined in Table 1.

We have developed an enclosure table (Figure 4) to provide details of the distribution and patterns of food access measures for each state (Figure 3). We have grouped the states by regions to facilitate decision making and other complex cognitive activities such as planning. Researchers can explore Figure 4 from diverse perspectives, including checking for common and unique patterns in the distribution of the counts of food access patterns. In Figure 4, we obtained additional details of the 16-digit food access pattern of “0011110111100111” (pattern with count of 139 in Figure 3). 

We observed 23 states with the pattern “0011110111100111”, with Arizona, Montana, New Mexico and Texas having double digit counts in a range of 1 to 16. U.S. Health and Human Services Regions 1 (Connecticut, Maine, Massachusetts, New Hampshire, Rhode Island and Vermont) and Regions 2 (New Jersey and New York) did not have census tracts described by the pattern “0011110111100111”. Within the 23 states, we also identified counties in nine states with more than one census tracts with the food access pattern “0011110111100111”. The states and associated counties are Alaska [Matanuska-Susitna]; Arizona [Apache, Cochise, Gila, Mohave]; Minnesota [St. Louis]; New Mexico [San Miguel]; Oregon [Klamath]; South Dakota [Bennett]; Texas [Uvalde]; Utah [San Juan]; and Washington [Clallam]. 

#### 3.2.3. Knowledge Visualization for Coordination of Environmental Protection Regions and Food Accessibility

**Figure 5:**
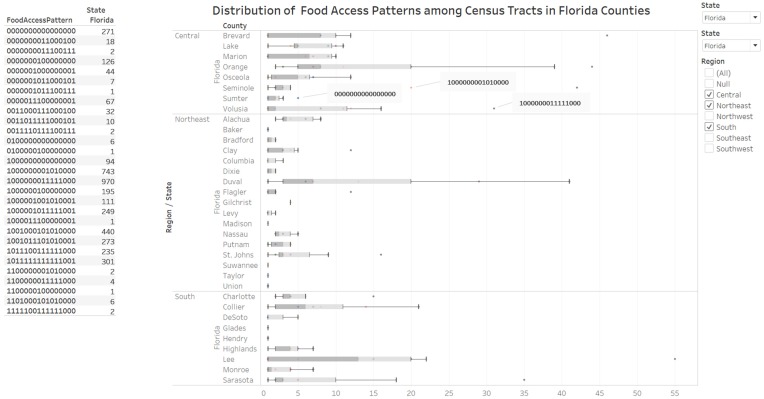
Distribution of food access patterns among census tracts in counties. The dashboard integrates the list of food access patterns and a box plot of the distribution of counts of census tracts with specific food access patterns. In this example, there are 29 food access patterns in Florida (recall function for knowledge visualization). The outlier patterns of food access indicate over-represented food access patterns. Census tracts with food access pattern 1000000011111000 (urban census tract flagged for low access variables) was over-represented (31 out of 113) in Volusia county of Florida.

**Figure 6:**
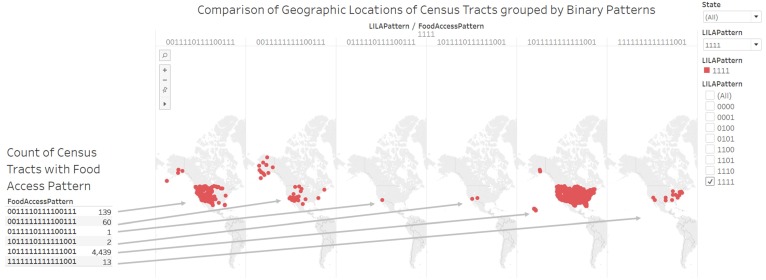
Comparison of geographic locations of census tracts grouped by binary patterns. In this example, there are six binary numbers that describe census tracts that have low income low access (attention function of knowledge visualization). The geographic map can support decision making on census tracts with specific food access pattern (motivation function of knowledge visualization).

The dashboard (Figure 5) integrates an enclosure table view listing the food access patterns for the selected state(s) with a box plot view of the count of food access patterns for counties in the state selected. The filters available allow the researcher to decide the state and group of counties to obtain additional details. We have also developed a dashboard that integrated an enclosure table and a geographic map (Figure 6). Figure 5 has a coordination function by integrating environmental protection regions and food accessibility for the State of Florida. The visualization demonstrates that 29 16-digit food access patterns describe the census tracts in Florida. The box-and-whisker plot (box plot) reveals food access patterns with a count of census tracts outside the upper whisker of the box plot. In Volusia County located in Central Florida, an outlier food access pattern (1000000011111000) has 31 urban census tracts of the 113 census tracts in the county that are not labeled as food deserts. We implemented an elaboration by interacting with the points on the box plot to identify 16 census tracts in Volusia County that are labeled as urban food deserts.

#### 3.2.4. Knowledge Visualization for Attention and Motivation on Locations of Low-Income Low-Access Census Tracts

In Figure 6, we have deployed a geographic map to elaborate on the six types of census tracts that are flagged for all the four LILA measures. The food access pattern 1011111111111001 describes the presence or absence of 16 food access measures for 4439 census tracts in the 50 states and the District of Columbia (DC). There were 13 urban census tracts labeled as food deserts and have group quarters. 

We observed 4654 census tracts designated as low-income low-access (LILA) and were described by six patterns: 0111110111100111 (1 census tract), 1011110111111001 (2 census tracts), 1111111111111001 (13 census tracts), 0011111111100111 (60 census tracts), 0011110111100111 (139 census tracts), and 1011111111111001 (4439 census tracts). The urban census tracts described by binary number 1111111111111001 are in counties located in Alabama, Arizona, Connecticut, Indiana, Maryland, Michigan, Nevada, New York, North Carolina, Ohio, Oklahoma and Texas (Table 4).

#### 3.2.5. Knowledge Visualization for New Insights on General Tract Characteristics of Census Tracts with Identical Food Access Pattern

**Figure 7:**
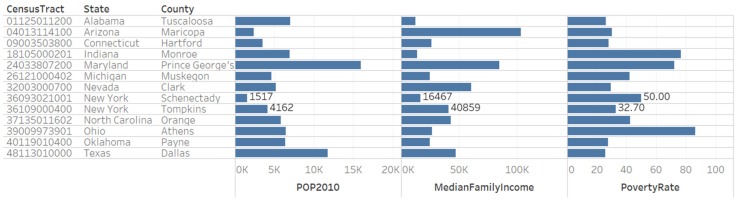
Comparison of population (2010), median family income and poverty rate for census tracts with food access pattern 1111111111111001 (not flagged from low-access tracts at 10 and 20 miles). The knowledge visualization provides new insights on the relationships that exists among these counties.

We designed a bar plot view to compare the three general tract characteristics (population (2010), median family income, and poverty rate) for the group of 13 census tracts with food access pattern 1111111111111001 (Figure 7). The estimates of median family income and poverty rate are from the 2010–2014 American Community Survey. The plot allowed us to compare the data for two census tracts in the state of New York.

#### 3.2.6. Knowledge Visualization for Attention to Food Accessibility in Rural Census Tracts

**Figure 8:**
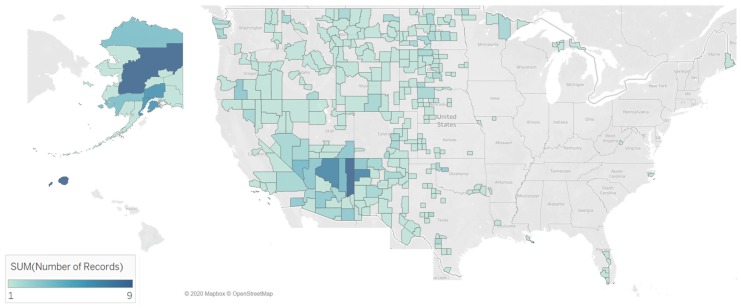
Geographic maps representing counties in the United States with census tracts designated as low access at 20 miles. A low-access tract at 20 miles is a rural tract with at least 500 people, or 33 percent of the population, living more than 20 miles from the nearest supermarket, supercenter or large grocery store. A function of this knowledge visualization is attention to the presence and location of counties. The color intensity is based on the number of records (census tracts) in the county flagged for low access at 20 miles.

According to the Food Access Research Atlas documentation, a low-access tract at 20 miles is a rural tract with at least 500 people, or 33 percent of the population, living more than 20 miles from the nearest supermarket, supercenter or large grocery store. Six food access patterns that define low-access tract at 20 miles and their census tract counts are 0111110111100111 (1 census tract); 0100000011100111 (3 census tracts); 0000001011100111 (7 census tracts); 0011111111100111 (60 census tracts); 0011110111100111 (139 census tracts); and 0000000011100111 (178 census tracts). We constructed a geographic map view to display the counties where low-access tract at 20 miles are located (Figure 3). Of the 388 low access at 20 miles census tracts, 245 (63%) are located in the states in the west of the United States: Alaska (30), Arizona (41), California (16), Colorado (16), Hawaii (1), Idaho (11), Montana (32), Nevada (15), New Mexico (33), Oregon (12), Utah (15), Washington (11), and Wyoming (12) (Figure 8).

### 3.3. Knowledge Visualizations to Inform Decision Making for Improving Food Accessibility and Reducing Adult Obesity Rates

**Figure 9:**
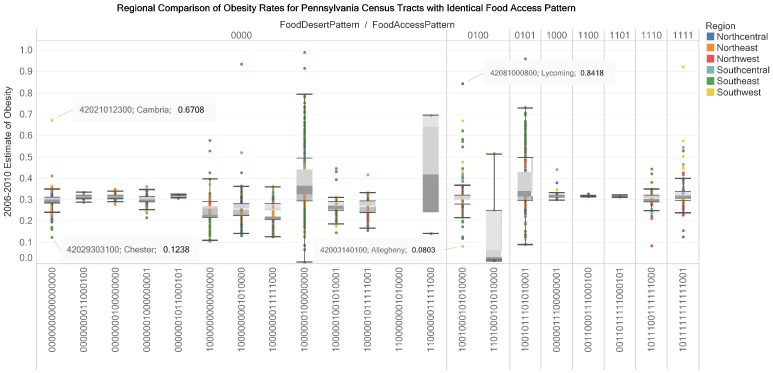
Regional comparison of estimates of obesity rates (2006–2010) for census tracts with the same food access pattern in Pennsylvania. We designed the box plot to support decisions on differences in obesity rates in regions. The design of the view allows one or more regions to be selected (coordination function of the knowledge visualization).

**Figure 10:**
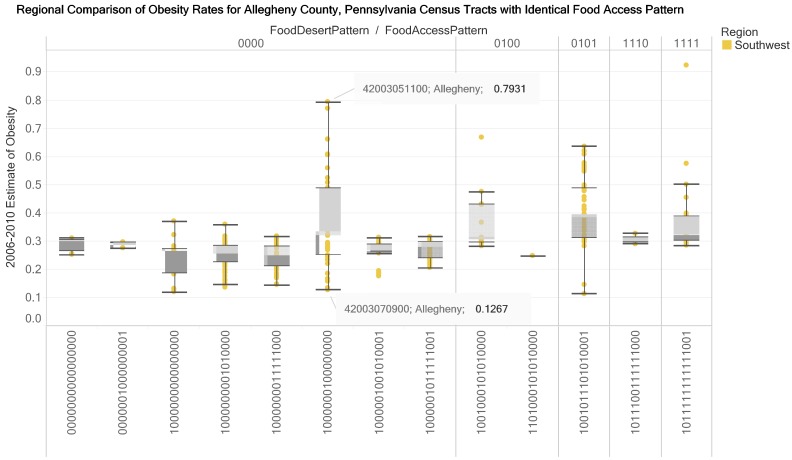
Regional comparison of estimates of obesity rates (2006–2010) for census tracts with the same food access pattern in Allegheny County, Southwest Pennsylvania. We designed the box plot to support decisions on differences in obesity rates in a county (attention and motivation functions of knowledge visualization). The design of the view allows one or more counties to be selected (elaboration function of the knowledge visualization).

**Figure 11:**
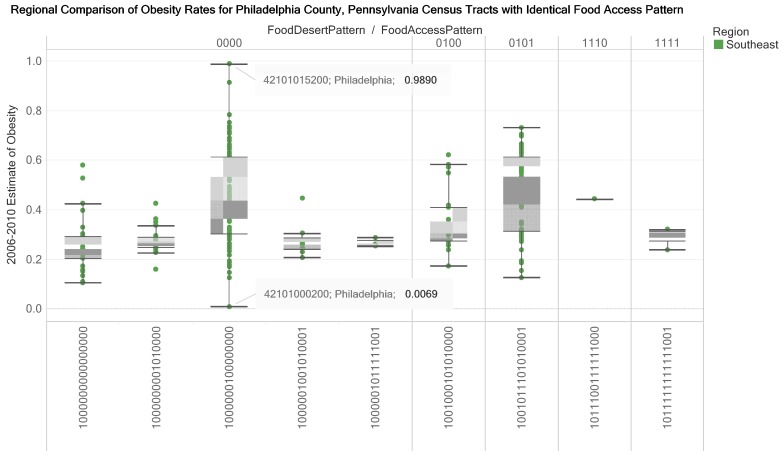
Regional comparison of estimates of obesity rates (2006–2010) for census tracts with the same food access pattern in Philadelphia County, Southeast Pennsylvania. We designed the box plot to support decisions on differences in obesity rates in a county (attention and motivation functions of knowledge visualization). The design of the view allows one or more counties to be selected (elaboration function of the knowledge visualization).

We assigned binary numbers describing food access patterns to 2620 census tracts in a dataset of estimates of obesity rates in adults (2006–2010) for 3121 census tracts in Pennsylvania. A box-and-whisker plot design represents data on LILA Patterns, food access patterns, region and 2006 to 2010 estimates of obesity rates (Figure 9 and Figure 10). In Figure 11, a bar plot of the obesity rates (coded by size) in a county for census tracts with a food pattern is connected to (1) a geographic map or (2) website with geographic map and demographic details of a census tract (Figure 11). The interactive visualizations provide benefits of coordination, attention, recall, motivation, elaboration and new insights. In Figure 9, the box-and-whisker plot (box plot) coordinates (1) the regional classification in Pennsylvania of census tracts; (2) adult obesity rates for census tracts and (3) the patterns of food access measures for census tracts. In this knowledge visualization, the 21 food access patterns are grouped into eight 4-digit LILA patterns with 13 food access patterns not classified as food deserts (Figure 9). Furthermore, none of the food access patterns are assigned as low-access tract at 20 miles (15th digit). 

The length of the whisker of the box-and-whisker plot (box plot) gives insights on the distribution of the estimated adult obesity rates for census tracts with the same food access pattern. The color of the census tracts encodes regional locations. The upper outlier and lower outlier values provide a guide to the identification of census tracts with significant differences in obesity rates. For example, census tract 42003140100 in Allegheny County (Southwest Pennsylvania) is compared to census tract 42081000800 in Lycoming County (Northcentral Pennsylvania). The visualization also provides a motivation to seek details on the reason(s) for the differences in the obesity rates. 

**Figure 12:**
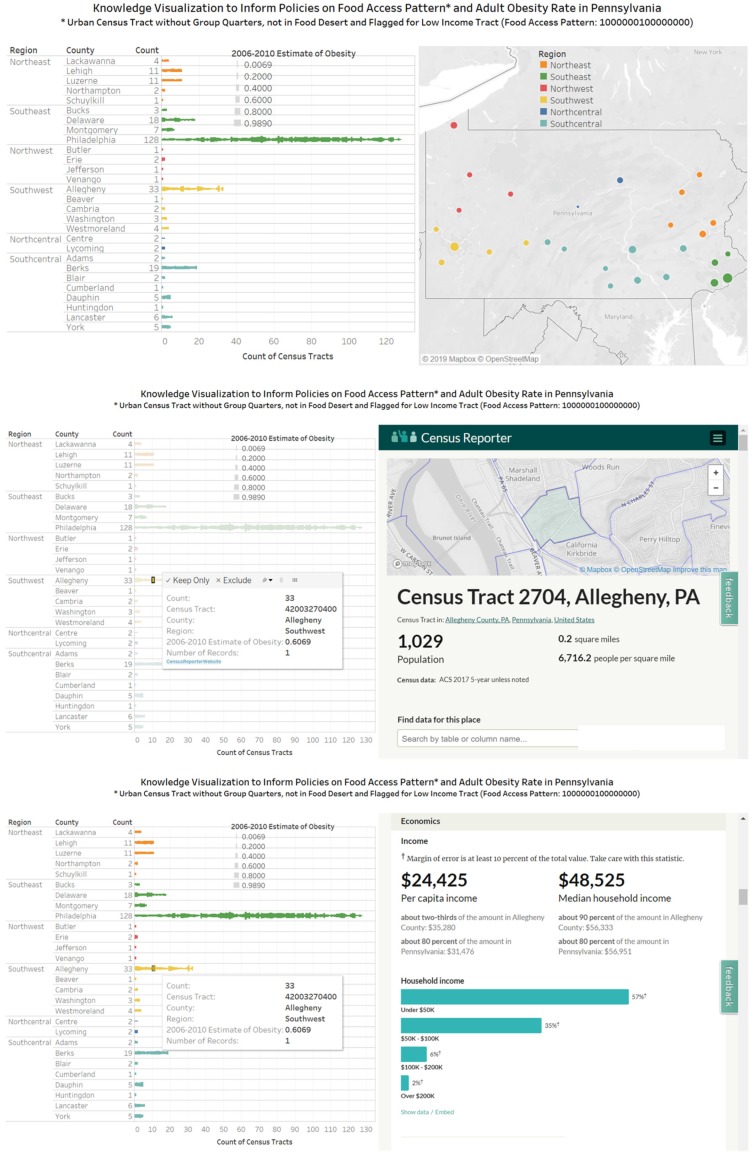
Bar plot of estimates of obesity rates (2006–2010) for census tracts with the same food access pattern and connected to geographic map or website. The design of the views allows for knowledge visualization benefits of coordination, attention, recall, motivation, elaboration and new insights.

The box plot in Figure 10 represents data for a county (Allegheny) and reveals that for food access pattern 1000000100000000 the estimated obesity rate for census tract 42003070900 is in the lower whisker while the estimated obesity rate for the census tract 42003051100 is in the upper whisker. For Pennsylvania’s Allegheny County, two of the thirteen 16-digit food access patterns are rural census tracts. A comparison box plot profile is presented for Philadelphia County. In the three visualizations (Figure 9, Figure 10 and Figure 11), the food access pattern 1000000100000000 (urban census tract without group quarters, not in food desert and flagged for low-income tract) has the longest whisker. In Figure 12, multiple views are presented for example interactions that could be made for a food access pattern and obesity rates for census tracts in a county. The top visualization coordinates the obesity rates with the count of census tracts in a county and the geographic location. The middle and bottom visualizations coordinate the bar plot and a web resource (the Census Reporter website). 

## 4. Discussion

### 4.1. Overview

The aim of this article is to promote the use of knowledge visualization frameworks in the creation and transfer of complex public health knowledge. We are developing a series of knowledge visualizations, which represent new knowledge and communicates complex data analytic results on the 18 May 2017 version of the Food Access Research Atlas (FARA) dataset. We recognized that datasets and visual representations from public health-relevant datasets must provide explanation (why) and prediction (what-if) to support priority setting and evaluation work of health system decision makers [15]. Thus, our research has produced a value-added Food Access Research Atlas (FARA) dataset. A value added is the binary number encoding of the 16 FARA food access measures for the 72,684 census tracts (Figure 2, Figure 3, Figure 4, Figure 5, Figure 6 and Figure 7; Table 1, Table 2, Table 3 and Table 4).

We then integrated the valued-added FARA dataset with a dataset on the 2006 to 2010 estimates of adult obesity rates in 2680 census tracts of the 67 counties of the Commonwealth of Pennsylvania. A value added to the obesity data is a regional context (e.g., obtained from the grouping of census tracts to environmental protection regions). The dataset from Pennsylvania presents a use case for the knowledge visualizations produced (Figure 9, Figure 10, Figure 11 and Figure 12). From a global perspective, our research aligns with the United Nations Sustainable Development Goal 2 (SDG-2) that aims to “End hunger, achieve food security and improved nutrition and promote sustainable agriculture” [47]. In summary, we have provided an approach for developing a value-added dataset from the FARA dataset combined with visualizations with underlying cognitive, emotional and social functions. The remainder of discussion section consists of the following sections (1) Knowledge Visualization as Integrated Explanation and Prediction System in Public Health; (2) Validation of the Accuracy of Value-Added Datasets; (3) Opportunities for Natural Language Processing of Large-Scale Text on Food Access; (4) Opportunities to Collect Primary Data on Rural Census Tracts on Factors Influencing Obesity Rates; (5) Opportunities for Census Tract Level Research on Nutritional Inequality and Nutrition Therapy in Diabetic Care; and (6) Limitations of Research and Strategies to address Limitations.

### 4.2. Knowledge Visualization as Integrated Explanation and Prediction System in Public Health

Based on our findings, we propose that knowledge visualization resources developed with public health-relevant datasets could be designed as integrated explanation and prediction system for public health research and practice. A major implication of our research is to inform decision making for improving food accessibility and reducing obesity rates in the United States [48,49]. The knowledge visualizations and other data products reported here could serve as data resources for community engagement and legislative actions on food access. The binary codes grouped the 72,864 census tracts into 55,172 urban census tracts and 17,692 rural census tracts with specific FARA food access measures for exploring mechanisms of obesity prevalence in working age adults [50].

We have designed and implemented the knowledge visualizations for explanation and prediction in health systems decision making to follow guidelines on the functions (coordination, attention, recall, motivation, elaboration, and new insights); and design principles of knowledge visualization [10,16]. Additionally, our research follows the frameworks for (1) interaction design for complex cognitive activities with visual representations [36,37]; (2) knowledge generation model for visual analytics [42]; and (3) transition of data to knowledge through information and evidence where evidence is relevant, robust, repeatable, and reproducible [13].

### 4.3. Validation of the Accuracy of Value-Added Datasets

Our first research objective was to design and implement knowledge visualizations that communicate census tracts in the FARA dataset as subgroups defined by binary numbers. Thus, we have developed a knowledge visualization (that combines an enclosure table and a bar plot) for attention and new insights on the distribution of the absence (0) or presence (1) of the 16 FARA food access measures (variables) in the 72,684 census tract dataset (Figure 2). The count of census tracts and counties reported in other publications helped to confirm the integrity of the binary number added FARA dataset. A publication on food deserts in the State of Texas confirmed our data of 5258 census tracts from 254 counties as well as 29 rural census tracts designated as low-access tract at 20 miles [51]. A research done at East Tennessee State University confirmed our dataset on the presence of 1497 census tracts in the State of Tennessee [52]. The count of census tracts per state in our analysis is identical to a data table in a May 2019 USDA Economic Research Service report titled “Understanding Low-Income and Low-Access Census Tracts Across the Nation: Subnational and Subpopulation Estimates of Access to Healthy Food” [8]. Additional evidence for the accuracy of the patterns is deduced from Figure 3, where the food access patterns for urban and rural census tracts starts with 1 and 0 respectively. The 15th digit number for all the urban food access patterns had a “0”. The 4-digit binary codes for the Low-Income Low-Access (LILA) census tracts makes it possible to predict 16 types of LILA census tracts. The 16-digit binary allows for 65,536 types of Food Access Patterns. This accuracy check as well as the possibility to classify census tracts into groups is consistent with the framework that the evidence to generate knowledge in public health informatics and data science must be relevant, robust, repeatable and reproducible [13].

### 4.4. Opportunities for Natural Language Processing of Large-Scale Text on Food Access

In Figure 3, the knowledge visualization displays a comparison of the count distribution of the 4-digit LILA (low-income low-access) patterns by rural and urban status. Using the pattern with the highest census tract count of 139 in the LILA pattern “1111”, there were 14 census tracts in 13 counties in Texas (i.e., Bandera, Briscoe, Cottle, Foard, Hall, Hudspeth, Real, Starr, Sterling, Terrell, Throckmorton, Uvalde and Zavala) while there were two census tracts in two Florida counties (Hendry and Okeechobee). This comparison led us to generate additional knowledge visualizations (Figure 4, Figure 5, Figure 6, Figure 7 and Figure 8) to provide diverse functions. The knowledge communicated include (1) the count of census tracts (Figure 4); (2) the regional placement of county-level food access patterns (Figure 5); (3) the geographic coordinates of food access patterns (Figure 6); (4) the median family income and poverty rate in a list of census tracts with identical food access patterns (Table 2, Figure 7); and (5) the geographic locations of counties of rural census tracts with at least 500 people, or 33 percent of the population, living more than 20 miles from the nearest supermarket, supercenter or large grocery store (Figure 8). Our methods for knowledge visualizations can be applied to professionally collected datasets in the May 2019 USDA ERS report on low-income low-access and low-access census tracts in the United States [8]. Furthermore, methods of artificial intelligence specifically natural language processing can be applied to the textual data (such as sentences, paragraphs, statements, words, table titles, figure captions) from public health-relevant reports [53,54,55,56]. A search of google scholar in December 2019 with the term “food access” returned 62,800 results (3410 results for 2019). Thus, a goal of natural language processing of large-scale text on food access includes promoting emergent knowledge creation and knowledge transfer necessary for knowledge-based team decision making [53,57].

### 4.5. Opportunities to Collect Primary Data on Rural Census Tracts on Factors Influencing Obesity Rates

We observed 388 census tracts described as low-access tract at 20 miles. Several factors predispose residents of rural areas in the United States to a higher risk of chronic diseases such as obesity compared to residents of urban areas [58,59]. Additionally, weight gain in rural areas is the main factor currently driving the global obesity epidemic [60]. Our analysis classified 17,692 census tracts as rural tracts described by 18 food access patterns (Figure 3). Our prior research on physical, social and economic access to fresh fruits and vegetables at farmers markets [55] followed the conceptual framework of the food environment, which is within the food system and based on the socio-ecological theory [2].

According to the conceptual framework of the food environment, the inputs to the food environment are from production, storage, transformation and transportation of food [2]. The food environment is depicted as the interface within the wider food system including dimensions in the external domain (availability, prices, vendor and product properties, marketing and regulation) and personal domain (accessibility, affordability, convenience, and desirability) that interact to shape people’s food acquisition and consumption. Research on the external domain and personal domain of the food environment in rural census tracts can help develop solutions to reduce obesity rates. Some recent publications that could be replicated or customized include assessment of nutrition environment [61,62], food environment for children [63], factors influencing food choices among older adults [64] and participation in food assistance programs for women, infants and children [65].

### 4.6. Opportunities for Census Tract Level Research on Nutritional Inequality and Nutrition Therapy in Diabetic Care

Of the 72,864 census tracts in the FARA dataset, 41,994 census tracts are low-income tracts (digit 8 of the 16 digit food access pattern) (Figure 2, a subset in Figure 6). We observed 21 food access patterns that are flagged for low-income census tract. Current on nutritional inequality in described “why the wealthy eat more healthfully than the poor in the United States” [66]. The findings proposes polices are needed to subsidize purchases of healthy foods by low-income households to reduce nutritional inequality [66]. The subgroups of census tracts defined by the food access patterns could serve as locations for research on the effects of increasing demand for healthy foods through price subsidies.

The knowledge visualizations in Figure 9, Figure 10, Figure 11 and Figure 12 communicates knowledge on food access patterns and obesity rates at geographic levels of regional, county, and census tracts. This multi-level approach could inform coordinated multi-level decision making for improving food accessibility and reducing chronic diseases in locations defined by food access measures. The availability of datasets on (1) food access measures for United States census tracts and (2) the 2006 to 2010 adult obesity rates for the Commonwealth of Pennsylvania have provided the input datasets needed for us to design and implement knowledge visualizations. As expanded and new data on obesity rates becomes available, the visualizations developed could be updated to reflect additional spatial or longitudinal trends. Obesity is a modifiable risk factor for type 2 diabetes [28], and there is consensus that nutrition therapy is an efficacious and cost-effective component of type 2 diabetic care [67]. Thus, we plan future data analytics to connect census tract level datasets on food access to census tract level diabetes-related hospital use and diabetes related complications [68,69].

### 4.7. Limitations of Research and Strategies to Address Limitations

The limitations and strengths of secondary data analysis could affect the results of our research [70]. The objectives for the data collected by the Food Access Research Atlas may not necessarily reflect the research objectives in this study. The data analyzed are historical datasets and the current situations in census tracts could be different. However, the food access patterns represent a national sample and could be useful for evaluating the changing patterns of the food access measures for census tracts in the FARA dataset. The metadata or statement regarding statistical or data transformation performed may not be available to authors of the secondary data analysis. For example, we were unable to determine whether the estimate of adult obesity rates is age adjusted. Since, we did not collect the data directly, we rely on a data-mediated knowledge of food access via the interactive visual representations we developed [71,72]. Thus, we have avoided constructing incorrect (mis-mediated) knowledge of food accessibility by combining the knowledge visualizations with our prior knowledge on food access and public health. Our discussion of the knowledge visualizations also provides examples of research that can stimulate the collection of primary data for groups of census tracts. From the knowledge visualization perspective, errors in the dataset will affect the representation and interpretation of the datasets. We have addressed this limitation through accuracy checks of the datasets and the use of knowledge visualization frameworks.

Our focus on interactive visualization makes it necessary for the use of online access (see supplementary materials section). We have developed the interactive visualizations in software that has a reader version that permits interaction with the knowledge visualizations. We have used approaches of working with common visualizations (such as box plots, bar plots and maps) helps to address the limitation of the visualizations being overwhelming or leading to overconfidence or bias in the interpretation of the insights [44,45]. This limitation stemming from uncommon visualizations also provides opportunities for designing learning experiences for understanding uncommon visualizations in public health [12]. Cognition of data is positively influenced by familiarity with the visuals used. Finally, our results could be affected by the limitations of the use of income and access measures for inferring food accessibility.

## 5. Conclusions

The aim of this article is to promote the use of knowledge visualization frameworks in creation and transfer of complex public health knowledge. Datasets of national public health relevance such as the Food Access Research Atlas (FARA) present opportunities for interactive data visualization techniques of cognitive, social and emotional benefits. We have designed and implemented knowledge visualizations that have the collective purpose of knowledge transfer and specific functions of attention, recall, elaboration, motivation, coordination, and motivation. The knowledge visualizations communicate knowledge on food access patterns and obesity rates age geographic levels of regional, county, and census tracts. This multi-level approach could inform coordinated multi-level decision making for improving food accessibility and reducing chronic diseases in locations defined by food access measures.

## Figures and Tables

**Table 1:** Variables in Food Access Research Atlas for constructing binary number for census tracts.

Field ^1^	LongName	Position in Binary Number
Urban	Urban tract	1
GroupQuartersFlag	Group quarters, tract with high share	2
LILATracts_1And10	Low income and low access tract measured at 1 mile for urban areas and 10 miles for rural areas	3
LILATracts_halfAnd10	Low income and low access tract measured at 1/2 mile for urban areas and 10 miles for rural areas	4
LILATracts_1And20	Low income and low access tract measured at 1 mile for urban areas and 20 miles for rural areas	5
LILATracts_Vehicle	Low income and low access tract using vehicle access or low income and low access tract measured at 20 miles	6
HUNVFlag	Vehicle access, tract with low vehicle access	7
LowIncomeTracts	Low income tract	8
LA1and10	Low access tract at 1 mile for urban areas and 10 miles for rural areas	9
LAhalfand10	Low access tract at 1/2 mile for urban areas and 10 miles for rural areas	10
LA1and20	Low access tract at 1 mile for urban areas and 20 miles for rural areas	11
LATracts_half	Low access tract at 1/2 mile	12
LATracts1	Low access tract at 1 mile	13
LATracts10	Low access tract at 10 miles	14
LATracts20	Low access tract at 20 miles	15
LATractsVehicle_20	Low access tract using vehicle access and at 20 miles in rural areas	16

^1^ Source: Food Access Research Atlas (FARA) [5,6].

**Table 2:** Percentage of census tracts in rural areas not flagged for food access measures (US HHS Regions 1 through Region 5).

US HHS Regions ^1^	State	Census Tract Count	All Zeros Count	Percent
Region 1	Connecticut	832	117	14.1
	Maine	355	150	42.3
	Massachusetts	1476	125	8.5
	New Hampshire	295	89	30.2
	Rhode Island	242	20	8.26
	Vermont	184	91	49.5
Region 2	New Jersey	2007	117	5.8
	New York	4907	446	9.1
Region 3	Delaware	218	27	12.4
	Maryland	1399	149	10.7
	Pennsylvania	3218	470	14.6
	Virginia	1900	188	9.9
	West Virginia	484	94	19.4
Region 4	Alabama	1179	194	16.5
	Florida	4214	271	6.4
	Georgia	1965	226	11.5
	Kentucky	1115	199	17.9
	Mississippi	662	71	10.7
	North Carolina	2192	382	17.4
	South Carolina	1103	152	13.8
	Tennessee	1497	280	18.7
Region 5	Illinois	3121	295	9.5
	Indiana	1508	334	22.2
	Michigan	2774	501	18.1
	Minnesota	1336	206	15.4
	Ohio	2949	440	14.9
	Wisconsin	1395	297	21.3

^1^ US HHS Regions: United States Health and Human Services Regions.

**Table 3:** Percentage of census tracts in rural areas not flagged for food access measures (US HHS Regions 6 through Region 10).

US HHS Regions ^1^	State	Census Tract Count	All Zeros Count	Percent
Region 6	Arkansas	686	130	19.0
	Louisiana	1143	111	9.7
	New Mexico	499	39	7.8
	Oklahoma	1046	158	15.1
	Texas	5258	489	9.3
Region 7	Iowa	825	225	27.3
	Kansas	770	112	14.6
	Missouri	1393	179	12.9
	Nebraska	532	77	14.5
Region 8	Colorado	1249	153	12.5
	Montana	271	56	20.7
	North Dakota	205	33	16.1
	South Dakota	222	38	17.1
	Utah	588	53	9.0
	Wyoming	132	20	15.2
Region 9	Arizona	1526	85	5.6
	California	8044	361	4.5
	Hawaii	332	33	9.9
	Nevada	687	40	5.8
Region 10	Alaska	167	29	17.4
	Idaho	298	49	16.4
	Oregon	830	121	14.6
	Washington	1455	176	12.1

^1^ US HHS Regions: United States Health and Human Services Regions.

**Table 4:** A group of low-income low-access census tracts in the Food Access Research Atlas.

Census Tract ^1^	State	County
01125011200	Alabama	Tuscaloosa
04013114100	Arizona	Maricopa
09003503800	Connecticut	Hartford
18105000201	Indiana	Monroe
24033807200	Maryland	Prince George’s
26121000402	Michigan	Muskegon
32003000700	Nevada	Clark
36093021001	New York	Schenectady
36109000400	New York	Tompkins
37135011602	North Carolina	Orange
39009973901	Ohio	Athens
40119010400	Oklahoma	Payne
48113010000	Texas	Dallas

^1^ Group of census tracts defined by 1111111111111001. The tracts are urban with group quarters and flagged for food access measures except for low-access tract at 10 miles (LATract10) and low-access tract at 20 miles (LATracts20).

## Supplementary Materials

The interactive versions of the tables and figures (knowledge visualizations) are available at https://public.tableau.com/profile/qeubic#!/vizhome/knowvizfoodaccess/abstract.
